# HNK-1+ cells in non-Hodgkin's lymphoma: lack of relation with transferrin receptor expression on malignant cells.

**DOI:** 10.1038/bjc.1985.26

**Published:** 1985-02

**Authors:** H. J. Schuurman, P. M. Kluin, G. C. de Gast, L. Kater

## Abstract

**Images:**


Br. J. Cancer (1985), 51, 171–177

### HNK-1$^+$ cells in non-Hodgkin's lymphoma: Lack of relation with transferrin receptor expression on malignant cells

H.-J. Schuurman[1,2], P.M. Kluin[2], G.C. de Gast[3] & L. Kater[1,2]

[1]*Division of Immunopathology, Department of Internal Medicine, and* [2]*Institute for Pathology, and* [3]*Division of Immunohaematology, Department of Haematology, University Hospital, Utrecht, The Netherlands.*

**Summary** It has been proposed that Natural Killer (NK) cell activity is involved in host defence against neoplasia, and that NK cells react with or recognize the transferrin receptor (TrR) on target cells. HNK-1 expression has been related to NK cell function. Therefore, in 118 cases of non-Hodgkin's lymphoma (NHL) we studied the occurrence and distribution of HNK-1$^+$ cells by immunohistochemistry, and simultaneously assessed the expression of TrR on malignant cells. In NHL of intermediate or high grade malignancy there was uniform expression of TrR on malignant cells. In low grade malignancy NHL, only lymphocytic and lymphoplasmacytoid lymphomas were TrR negative, except for faint staining of proliferation centres. In 23 cases of follicular lymphoma, 9 showed the absence of HNK-1$^+$ cells in neoplastic follicles. In 16/23 cases HNK-1$^+$ cells were present around follicles or in interfollicular areas: 8 of these cases revealed a higher density of HNK-1$^+$ cells at this site than inside the follicles. In 22/26 cases with high grade malignancy NHL, HNK-1$^+$ cells were absent or present in small density, which is different from the presence in higher density in low grade malignancy NHL. We conclude that (i) TrR expression on NHL cells is not obligatory related with histological class or malignancy grade of the tumour, and that (ii) HNK-1$^+$ cells are not universally present in areas of malignant cells, in particular in follicular lymphoma and in NHL of high grade malignancy.

Natural killer (NK) cells, being large granular mononuclear cells with characteristics of T lineage lymphocytes, are able to lyse tumour cells, virus-infected cells and undifferentiated normal cell types without prior sensitization *in vitro* (Herberman, 1983; Roder & Pross, 1982). These cells may play a potential role in host defence against tumours and resistance to metastasis. Most NK cells are detected by the monoclonal antibody HNK-1 (Leu 7) (Abo & Balch, 1981). In various lymphoid organs the numbers of HNK-1$^+$ cells correlate with NK activity (Roder & Pross, 1982). However, the HNK-1$^+$ cell population exerts heterogeneity (Abo *et al.*, 1983; Fast *et al.*, 1983; Lanier & Loken, 1984). HNK-1$^+$ cells which coexpress T lymphocyte markers form the major part of NK cells in adult bone marrow and lymph nodes. In contrast, in spleen and peripheral blood a significant population of HNK-1$^+$ cells lacks T lymphocyte markers, but reveals expression of the myeloid antigen M1; this subpopulation shows a higher NK activity than that with T cell markers (Abo *et al.*, 1983).

There seems to be no single target cell specificity of NK cells (Phillips *et al.*, 1980). It has been claimed that the transferrin receptor (TrR) is a target cell structure (Vodinelich *et al.*, 1983) or is involved in the recognition process, but this has recently been disputed (Dokhélar *et al.*, 1984). TrR is present on all metabolically active, proliferating cells (Trowbridge & Omary, 1981). By the interaction between NK cells and TrR, NK cells may regulate proliferation and expansion of cell clones, both in physiology and pathology (James & Ritchie, 1984).

In the normal lymph node, the localization of HNK-1$^+$ cells is restricted to germinal centres of secondary follicles (Hsu *et al.*, 1983; Ritchie *et al.*, 1983; Si & Whiteside, 1983), in coexpression with T cell markers (Banerjee & Thibert, 1983; Poppema *et al.*, 1983; Porwit-Ksiazek *et al.*, 1983). These areas are distinguished from other sites in the lymph node by a confluent presence of TrR expressing lymphocytes.

For non-Hodgkin's lymphoma (NHL) there are some data on the presence and localization of HNK-1$^+$ cells (Banerjee & Thibert, 1983; Poppema *et al.*, 1983; Porwit-Ksiazek *et al.*, 1983; Si & Whiteside, 1983; Swerdlow & Murray, 1984). With regard to TrR expression, a correlation has been reported between the percentage of TrR expressing cells in suspension of NHL tissue specimens and histological class of the tumour or clinical outcome of the disease (Habeshaw *et al.*, 1983; Kvaløy *et al.*, 1984). In a first evaluation of the putative role of NK activity to autologous tumour cells in NHL, we did an immunohistochemical study on the presence and distribution of HNK-1$^+$ cells in a large series of NHL of different histological classes. As the

Correspondence: H.-J. Schuurman.
Received 8 August 1984; and in revised form 3 October 1984.

© The Macmillan Press Ltd., 1985

TrR is suggested to represent a target cell structure or recognition structure for NK cells, the presence of this receptor on pathological cells was simultaneously investigated, and correlated with the presence and distribution of HNK-$1^+$ cells.

### Materials and methods

### *Lymph nodes*

The study included 118 NHL specimens presented for histopathological diagnosis to the Institute for Pathology. The routine analysis included histopathology, cytology, frozen tissue section analysis in immuno- and enzyme-histochemistry, and electron-microscopy (described in detail elsewhere, Kluin *et al.*, manuscript submitted). Each case was classified according to the Kiel classification (Lennert & Mohri, 1978). NHL, intermediate lymphocytic, was added: in the Kiel classification this entity forms a part of NHL, centrocytic. Immunohistochemical typing included application of monoclonal anti-T lymphocyte antibodies of the Leu series (Leu 1, Leu 2, Leu 3, Leu 5, Becton Dickinson, Mountain View, Calif., USA) and OKT 6 (Ortho Diagnostic Systems Inc., Raritan, NJ, USA), and anti-B cell antibodies B1 (Coulter Clone, Luton, UK), BA-1 (Hybritech Inc., San Diego, Calif., USA) or DAKO-pan-B (Dakopatts, Copenhagen, Denmark). These were applied in immunoperoxidase techniques (see below). In addition, anti-immunoglobulin light and heavy chain antisera conjugated to fluorescein isothiocyanate or tetramethyl rhodamine isothiocyanate were applied in (double) immunofluorescence (antisera from Behringwerke, Marburg-Lahn, W-Germany, Dakopatts or Kallestad, Austin, TX, USA). The NHL cases are presented in Table I. As control 10 lymph node specimens with normal histopathology were investigated.

### *Immunohistochemistry for HNK-$1^+$ cells and expression of TrR*

Part of the specimen was snap frozen and stored at temperatures below −70°C. Cryostat sections of 4–6 μm thickness were air-dried and fixed in acetone for 10 min at room temperature, followed by rinsing in PBS, pH 7.4. In immunoperoxidase staining a two-step procedure was followed: (1) incubation with antibody to HNK-1 (Leu 7, Becton Dickinson, diluted 1:50) or to TrR (B3/25, Hybritech, diluted 1:100) in PBS with 1% (wt/vol) human serum albumin (Behringwerke); (2) incubation with horseradish peroxidase-conjugated rabbit immunoglobulin to mouse immunoglobulin (Dakopatts), diluted 1:40 in PBS with 2.5% human AB serum. The staining was performed with 3-amino-9-ethylcarbazole (Aldrich Chemical Co., Milwaukee, WI, USA) and $H_2O_2$ in 0.1 M acetate buffer, pH 4.6 (Graham *et al.*, 1965). Sections were embedded in an aqueous solution of gelatin (18% wt/vol), glycerin (50% vol/vol) and phenol (1% wt/vol). In photography, a blue filter was applied.

The expression of TrR was scored as absent (−) or present (+). The occurrence of HNK-$1^+$ cells was scored as absent (−), scattered in low density (+, between ~50 and 200 $mm^{-2}$) or in moderate to high density (++, between ~200 and 1000 $mm^{-2}$ or more). In addition the tissue localization was evaluated.

### Results

### *Normal lymph node*

In all 10 cases investigated, there was a confluent occurrence of TrR expressing lymphocytes in the germinal centres of secondary follicles: primary follicles and follicle mantle zones were negative. In paracortical areas TrR expression was observed on nonlymphoid cells and possibly also on a few lymphocytes (Figure 1a). HNK-$1^+$ cells were found almost exclusively in the germinal centres, in most cases in moderate to high density (Figure 1b). Dependent on the sectioning of specimens there appeared to be an uneven distribution, the most concentrated areas being the cortical pole of the follicle. This distribution paralleled that of T lymphocytes (of T-helper phenotype) in the germinal centre. Primary follicles, mantle zones of secondary follicles and paracortical areas were almost negative.

### *Non-Hodgkin's lymphoma*

In histopathology, 42 cases were diagnosed as NHL of low grade malignancy (all with B cell phenotype), 50 as NHL of intermediate grade malignancy (all with a diffuse pattern; 49 with B cell phenotype and one without T or B cell markers (denoted non-T non-B)) and 26 as NHL of high grade malignancy (17 with T cell, 7 with B cell and 2 without T or B cell markers) (Table I). In the majority of cases TrR expression was found on malignant cells: in the first group 4 cases were found negative, and in the second group 5 cases. However, between the individual groups the distribution differed markedly. In NHL of low malignancy grade, 8/10 lymphocytic cases showed foci of positive cells, other cells being negative. Similarly, 4/9 lymphoplasmacytoid immunocytoma cases revealed foci of TrR positive cells, whereas in the other cases a diffuse presence of TrR positive cells was found. In 22/23 follicular CBCC cases TrR expression was found in neoplastic follicles. In 50 NHL cases of intermediate grade malignancy, a diffuse presence of TrR positive cells was found in 42 cases, whereas foci of TrR expressing cells were observed in 3 cases. All 26 cases of high grade malignancy showed TrR expression on cells at diffuse location. Representative examples of TrR expression on NHL cells are presented in Figure 1c, e, g and i).

HNK-1$^+$ cells were found in about two-thirds of NHL specimens: in low grade malignancy NHL 10/42 cases were negative, in intermediate grade malignancy NHL this was 18/50 cases and in high malignancy grade NHL this was 8/26 cases (Table I).

However, in NHL of low grade malignancy there was an uneven distribution. This phenomenon was most pronounced in CBCC follicular NHL. Out of 23 cases, 16 cases revealed HNK-1$^+$ cells around neoplastic follicles or in interfollicular areas. In 14 cases HNK-1$^+$ cells were found in neoplastic follicles. A predominant occurrence of HNK-1$^+$ cells around neoplastic follicles or in the the interfollicular area was observed in 8 cases, and 7 cases showed a predominance in follicles. In 4 cases the density of HNK-1$^+$ cells was similar outside and inside follicles. Four cases were negative for HNK-1$^+$ positive cells.

In NHL of intermediate grade malignancy, HNK-1$^+$ cells, if present (32/50 cases), were almost always observed in a diffuse distribution: in only one case an uneven distribution was observed, and in this case HNK-1$^+$ cells were observed in areas with high numbers of T lymphocytes. HNK-1$^+$ cells were observed in 18/26 cases of high grade malignancy NHL: all cases showed a diffuse

**Table I** Expression of transferrin receptor on malignant cells and occurrence and distribution of HNK-1$^+$ cells in non-Hodgkin's lymphoma[a]

| *Histopathological diagnosis* | *n* | *T* | *B* | *non-T non-B* | *Transferrin receptor* − | *focal* | *diffuse* | *HNK-1 positive cells* − | *focal* + | *focal* ++ | *diffuse* + | *diffuse* ++ |
|---|---|---|---|---|---|---|---|---|---|---|---|---|
| *Low grade malignancy* | | | | | | | | | | | | |
| lymphocytic | 10 | | 10 | | 2 | 8 | | | 3 | 5 | 2 | |
| lymphoplasmacytoid immunocytoma | 9 | | 9 | | 1 | 4 | 4 | 1 | 1 | 3 | 2 | 2 |
| CBCC follicular[b] | 23 | | 23 | | 1 | 22 | | 9 | 4 | 10 | | |
| Total | 42 | | 42 | | 4 | 34 | 4 | 10 | 8 | 18 | 4 | 2 |
| *Intermediate grade malignancy* | | | | | | | | | | | | |
| CBCC diffuse | 16 | | 15 | 1 | 2 | | 14 | 4 | | | 10 | 2 |
| CC intermediate | 10 | | 10 | | 1 | 1 | 8 | 1 | | 1 | 1 | 7 |
| lyphocytic | 8 | | 8 | | 2 | 2 | 4 | 4 | | | 4 | |
| CB diffuse | 15 | | 15 | | | | 15 | 9 | | | 5 | 1 |
| polymorphic immunocytoma | 1 | | 1 | | | | 1 | | | | 1 | |
| Total | 50 | | 49 | 1 | 5 | 3 | 42 | 18 | | 1 | 21 | 10 |
| *High grade malignancy* | | | | | | | | | | | | |
| immunoblastic | 16 | 7 | 7 | 2 | | | 16 | 2 | | | 10 | 4 |
| T lymphoblastic | 10 | 10 | | | | | 10 | 6 | | | 4 | |
| Total | 26 | 17 | 7 | 2 | | | 26 | 8 | | | 14 | 4 |

[a]The occurrence of HNK-1$^+$ cells is presented as absent (−), scattered in low density (+, between ~50 and 200 mm$^{-2}$) or in moderate to high density (++, between ~200 and 1000 mm$^{-2}$ or higher). Abbreviations: CBCC, centroblastic/centrocytic; CC, centrocytic; CB, centroblastic.

[b]The occurrence of HNK-1$^+$ cells in CBCC follicular is presented only for follicles. Around neoplastic follicles and in interfollicular areas the presence was: −, 7; +, 5; ++, 11. HNK-1$^+$ cells were absent in 4 cases, in 7 cases the density in neoplastic follicles was higher than around or between follicles, in 8 cases the reverse distribution was found, and in 4 cases the density in follicles was about similar to that around or between follicles.

(a)
(b)
(c)
(d)
(e)
(f)

**Figure 1** Expression of transferrin receptor (TrR) on malignant cells (left figures, *a, c, e, g, i*) and presence of HNK-1$^+$ cells (right figures, *b, d, f, h, j*) in corresponding areas of tissue sections from normal lymph node and non-Hodgkin's lymphoma. *a, b*; in normal lymph node, germinal centre cells of secondary follicles show TrR expression; in this area there are HNK-1$^+$ cells in moderate to high density ($\sim 500\,mm^{-2}$). *c, d*; in a case of lymphocytic (diffuse) NHL there is no TrR expression on malignant cells except for some foci, and HNK-1$^+$ cells are present in low density ($\sim 150\,mm^{-2}$). *e, f, g, h*; in CBCC follicular NHL there is TrR expression on malignant cells in follicles, and HNK-1$^+$ cells in moderate to high density are found for the first case (*f*) in the follicles ($\sim 750\,mm^{-2}$), for the second case (*h*) around follicles and in interfollicular areas ($\sim 500\,mm^{-2}$). *i, j*; in a case of T lymphoblastic NHL there is TrR expression on malignant cells at diffuse location, and HNK-1$^+$ cells are present in low density ($\sim 100\,mm^{-2}$).

distribution of HNK-1$^+$ cells. Representative examples of the occurrence and distribution of HNK-1$^+$ cells are presented in Figure 1d, f, h and j.

Concerning areas with mainly malignant cells, HNK-1$^+$ cells in moderate to high density were observed in 20/42 low grade malignancy NHL, in 11/50 intermediate grade malignancy NHL, and 4/26 high grade malignancy NHL. HNK-1$^+$ cells in low density were found in 12, 21 and 14 cases, respectively. There was no relation between the density of HNK-1$^+$ cells and T or B cell phenotype of the tumour cells.

### Discussion

In accord with TrR expression on germinal centre cells of secondary follicles in normal lymph nodes (Figure 1a), we found TrR positivity on almost all germinal centre cell derived malignant lymphocytes (i.e., CBCC follicular, CB and CC diffuse, Table I, Figure 1e and 1g). In these cases TrR positivity is mainly found in the malignant nodules. Other lymphoid tumours also showed TrR expression. Only NHL, lymphocytic and lymphoplasmacytoid immunocytoma, were TrR negative except for foci of TrR expressing cells (Table I, Figure 1c): these foci probably represent proliferation centres. We conclude that, except for these two NHL subtypes of low grade malignancy, the expression of TrR on malignant cells is not related to histological class or malignancy grade of the tumour.

For the percentage of TrR positive cells in cell suspension of NHL tissue specimens such a relationship has been reported (Habeshaw *et al.*, 1983; Kvaløy *et al.*, 1984). There are two explanations for this discrepancy between cell suspension and tissue section analysis:

— non-malignant cells, which do not express TrR, contribute to results of cell suspension analysis: the number of these cells may be large in cases of follicular lymphoma of low grade malignancy (Kluin *et al.*, manuscript submitted).
— diffuse lymphomas of low grade malignancy (lymphocytic and lymphoplasmacytoid immunocytoma) are TrR negative, except for foci mentioned above.

In a first evaluation of a putative role for NK cell activity to autologous tumour cells in NHL, we investigated the occurrence and distribution of HNK-1$^+$ cells by immunohistochemistry. The results do not indicate such a putative involvement. In NHL of high grade malignancy, most cases (22/26) revealed the absence or presence in low density of HNK-1$^+$ cells: in NHL of low or intermediate grade malignancy generally a larger density was found. This occurrence apparently parallels that of non-malignant lymphocytes mentioned above. Moreover, the results in follicular lymphomas can be considered. In contrast to normal lymph nodes, NHK-1$^+$ cells were observed around pathological follicles or in interfollicular areas in 16/23 cases, and in 8 cases the density around follicles exceeded that inside the neoplastic follicles (Figure 1h). Only in 10 of the cases did the neoplastic follicles reveal HNK-1$^+$ cells in moderate to high density similar to normal lymph node follicles. These observations are in partial discordance with preliminary data from the literature that follicular areas of nodular NHL contain HNK-1$^+$ cells (Banjeree & Thibert, 1983; Poppema *et al.*, 1983; Porwit-Ksiazek *et al.*, 1983). However, the presence of HNK-1$^+$ positive cells mainly around nodules has been noted for some cases of follicular lymphoma (Porwit-Ksiazek *et al.*, 1983; Si & Whiteside, 1983), e.g. for 4/10 cases in the series investigated by Swerdlow & Murray (1984). In agreement with our data, Banerjee & Thibert (1983) and Swerdlow & Murray (1984) have observed a low density of HNK$^+$ cells in NHL of high grade malignancy: this may be due to a dilution effect reflecting a low number of residual normal cells.

Our observations indicate that HNK-1$^+$ cells are not universally present in areas of malignant cells in NHL, in particular follicular NHL and NHL of high grade malignancy. In contrast to normal lymph node germinal centres, we found no indications for the presence of HNK-1$^+$ cells adjacent to TrR positive cells in lymphoid malignancies: this finding does not support a putative anti-tumour role of HNK-1$^+$ cells in non-Hodgkin's lymphoma. It remains to be established whether the presence of HNK-1$^+$ cells around pathological follicles or in interfollicular areas points to that role, i.e. an involvement in keeping follicular lymphomas localized, or merely reflects the remaining lymph node architecture.

The authors gratefully acknowledge the technical assistance by Mr J.G.N. Geertzema, and discussions with Prof. Dr J.A.M. van Unnik in the design of this study.

### References

ABO, T. & BALCH, C.M. (1981). A differentiation antigen of human NK and K cells identified by a monoclonal antibody (HNK-1). *J. Immunol.*, **127**, 1024.

ABO, T., MILLER, C.A., GARTLAND, G.L. & BALCH, C.M. (1983). Differentiation stages of human natural killer cells in lymphoid tissues from fetal to adult life. *J. Exp. Med.*, **157**, 273.

BANERJEE, D. & THIBERT, R.F. (1983). Natural killer-like cells found in B-cell compartments of human lymphoid tissues. *Nature*, **304**, 270.

DOKHËLAR, M.C., GARSON, D., TESTA, U. & TURSZ, T. (1984). Target structure for natural killer cells: evidence against a unique role for transferrin receptor. *Eur. J. Immunol.*, **14**, 240.

FAST, L.D., BEATTY, P., HANSEN, J.A. & NEWMAN, W. (1983). T cell nature and heterogeneity of recognition structures of human natural killer (NK) cells. *J. Immunol.*, **131**, 2404.

GRAHAM, R.C., LUNDHOLM, U. & KARNOVSKY, M.J. (1965). Cytochemical demonstration of peroxidase activity with 3-amino-9-ethylcarbazole. *J. Histochem. Cytochem.*, **13**, 150.

HABESHAW, J.A., LISTER, T.A., STANSFELD, A.G. & GREAVES, M.F. (1983). Correlation of transferrin receptor expression with histological class and outcome in Non-Hodgkin Lymphoma. *Lancet*, **i**, 498.

HERBERMAN, R.B. (1983). Possible role of natural killer cells in host resistance against tumours and other diseases. *Clin. Immunol. Allergy*, **3**, 479.

HSU, S.-M., COSSMAN, J. & JAFFE, E.S. (1983). Lymphocyte subsets in normal human lymphoid tissues. *Am. J. Clin. Pathol.*, **80**, 21.

JAMES, K. & RITCHIE, W.S. (1984). Do natural killer cells regulate B-cell activity? *Immunol. Today*, **5**, 193.

KVALØY, S., LANGHOLM, R., KAALHUS, O. & 4 others. (1984). Transferrin receptor and B-lymphoblast antigen – their relationship to DNA synthesis, histology and survival in B-cell lymphomas. *Int. J. Cancer*, **33**, 173.

LANIER, L.L. & LOKEN, M.R. (1984). Human lymphocyte subpopulations identified by using three-color immunofluorescence and flow cytometry analysis: correlation of Leu-2, Leu-3, Leu-7, Leu-8, and Leu-11 cell surface antigen expression. *J. Immunol.*, **132**, 151.

LENNERT, K. & MOHRI, N. (1978). Histopathology and diagnosis of non-Hodgkin's lymphomas. In: *Malignant Lymphomas Other Than Hodgkin's Disease*. (Ed. Lennert), Berlin: Springer Verlag, p. 111.

PORWIT-KSIAZEK, A., KSIAZEK, T. & BIBERFELD, P. (1983). Leu7$^+$ (HNK-1$^+$) cells. I. Selective compartmentalization of Leu7$^+$ cells with different immunophenotypes in lymphatic tissues and blood. *Scand. J. Immunol.*, **18**, 485.

PHILLIPS, W.H., ORTALDO, J.R. & HERBERMAN, R.B. (1980). Selective depletion of human natural killer cells on monolayers of target cells. *J. Immunol.*, **125**, 2322.

POPPEMA, S., VISSER, L. & DE LEIJ, L. (1983). Reactivity of presumed anti-natural killer cell antibody Leu 7 with intrafollicular T lymphocytes. *Clin. Exp. Immunol.*, **54**, 834.

RITCHIE, A.W.S., JAMES, K. & MICKLEM, H.S. (1983). The distribution and possible significance of cells identified in human lymphoid tissue by the monoclonal antibody HNK-1. *Clin. Exp. Immunol.*, **51**, 439.

RODER, J.C. & PROSS, H.F. (1982). The biology of the human natural killer cell. *J. Clin. Immunol.*, **2**, 249.

SI, L. & WHITESIDE, T.L. (1983). Tissue distribution of human NK cells studied with anti-Leu-7 monoclonal antibody. *J. Immunol.*, **130**, 2149.

SWERDLOW, S.M. & MURRAY, L.J. (1984). Natural killer (Leu 7+) cells in reactive lymphoid tissues and malignant lymphomas. *Am. J. Clin. Pathol.*, **81**, 459.

TROWBRIDGE, I.S. & OMARY, M.B. (1981). Human cell surface glycoprotein related to cell proliferation is the receptor for transferrin. *Proc. Natl Acad. Sci.*, **78**, 3039.

VODINELICH, L., SUTHERLAND, R., SCHNEIDER, C., NEWMAN, R. & GREAVES, M. (1983). Receptor for transferrin may be a "target" structure for natural killer cells. *Proc. Natl Acad. Sci.*, **80**, 835.


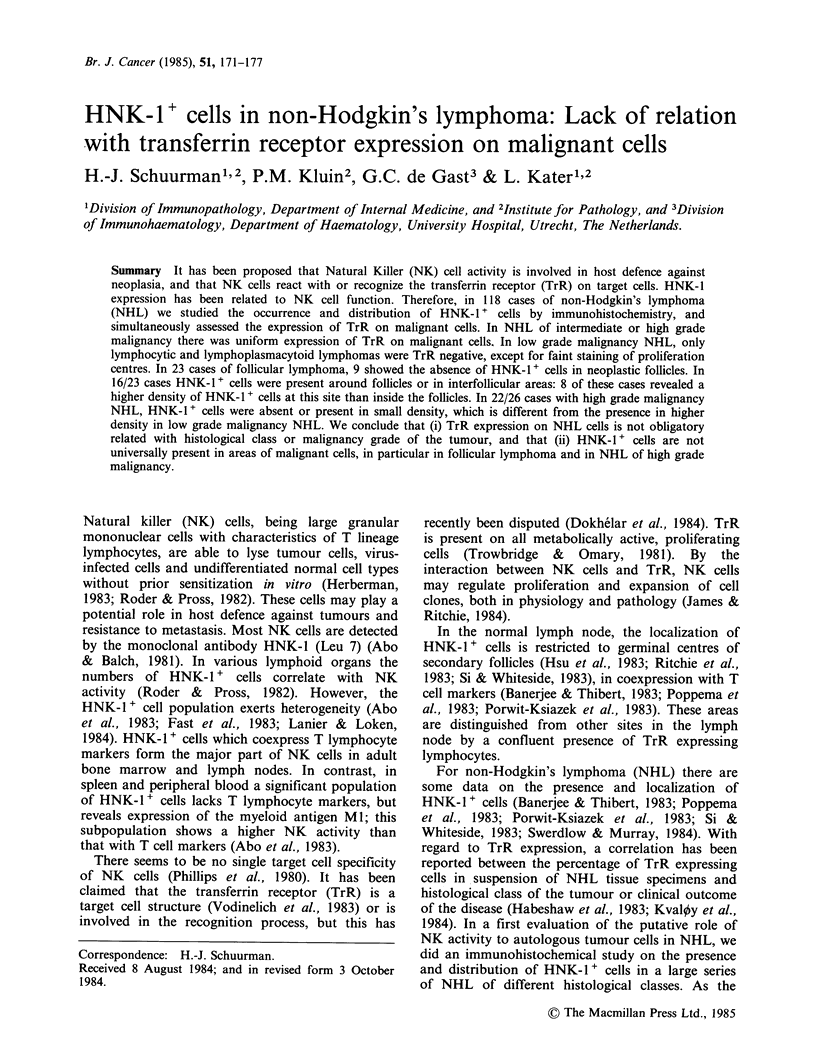

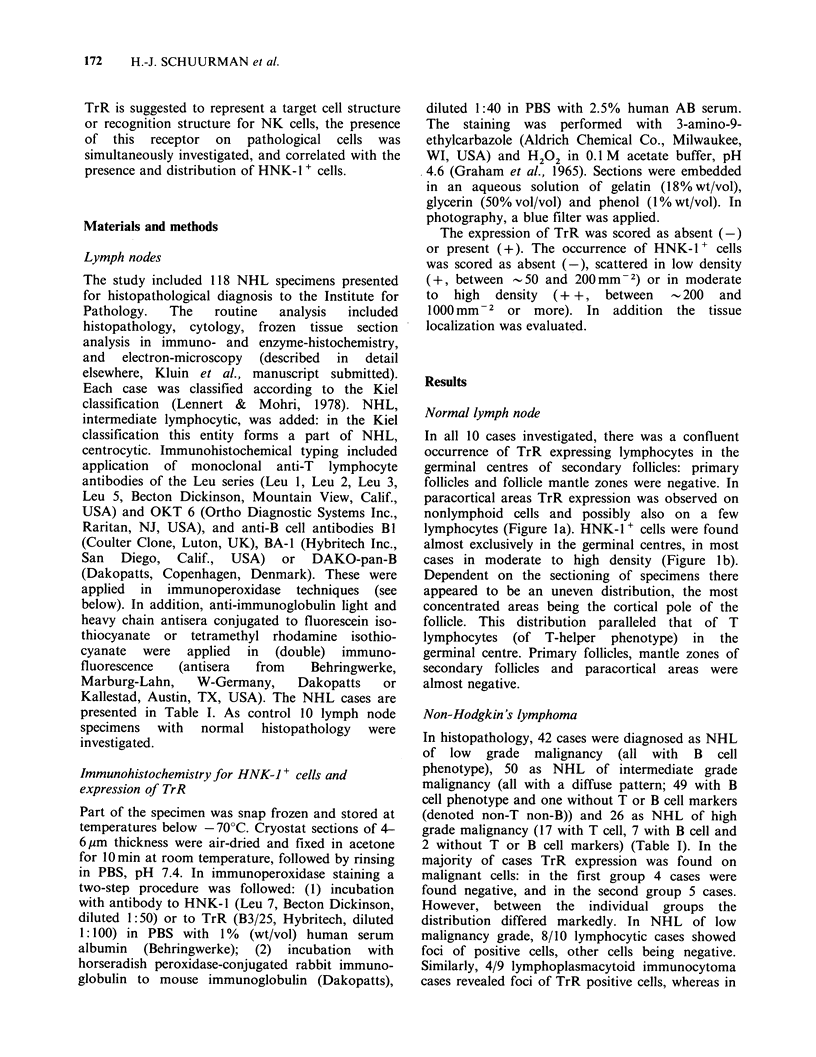

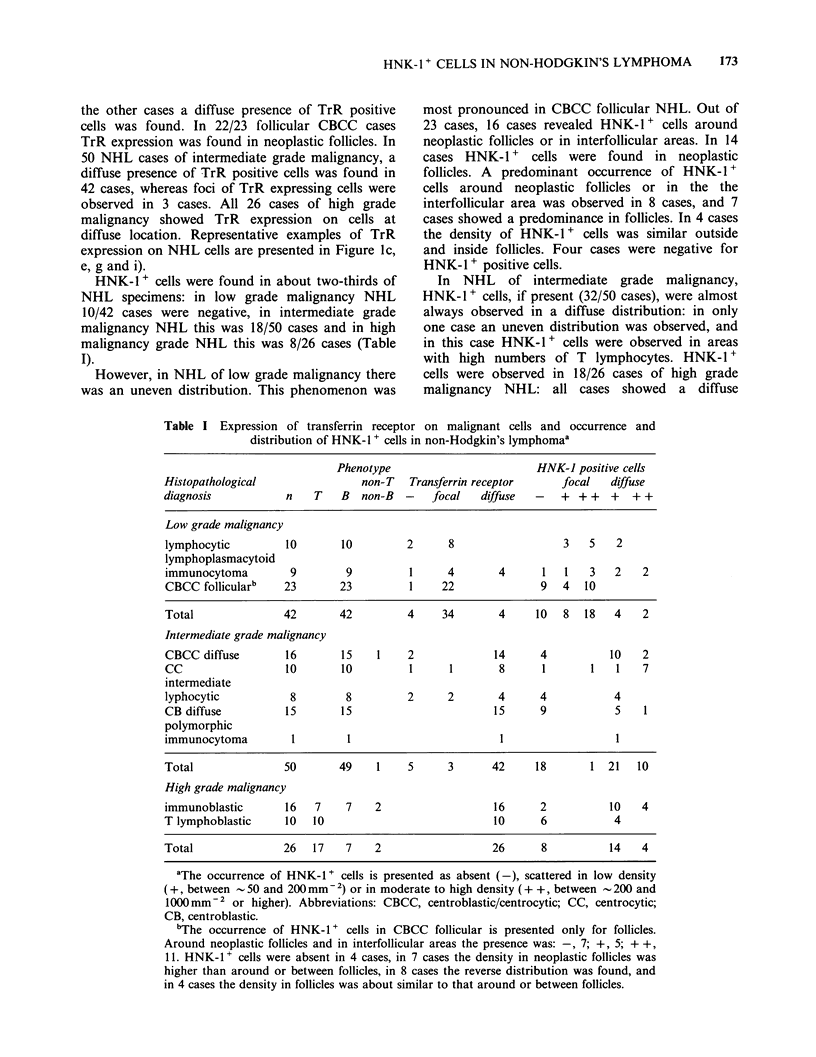

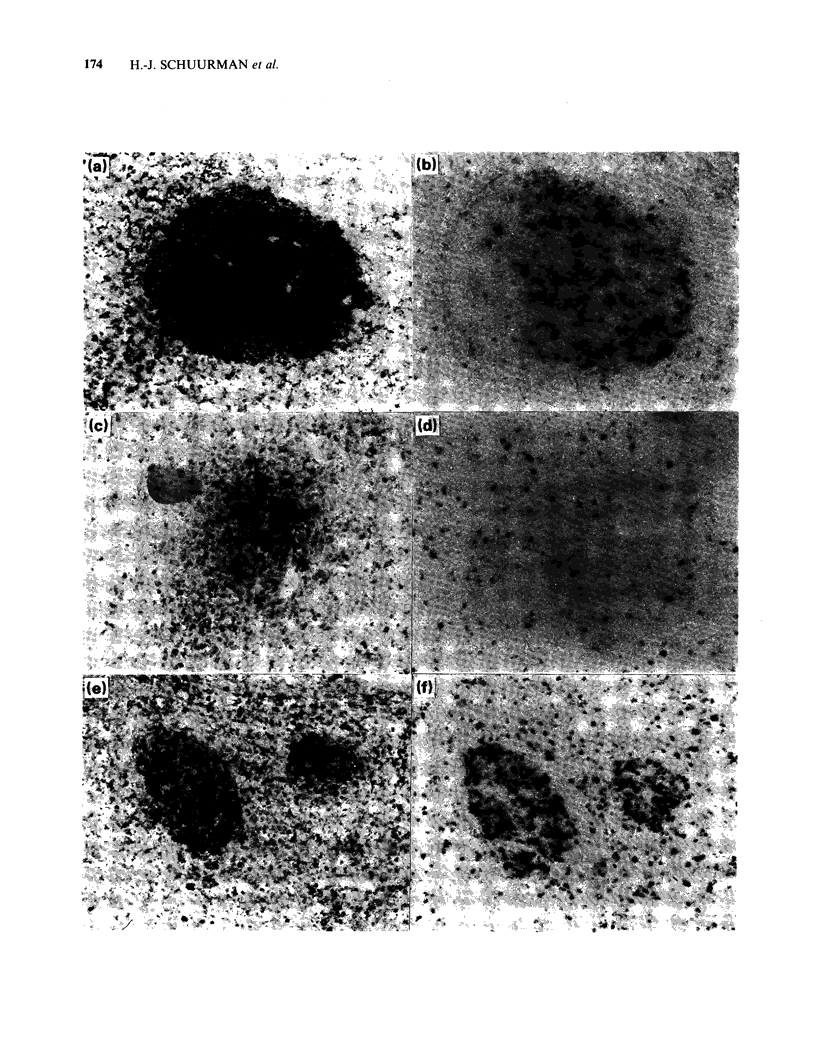

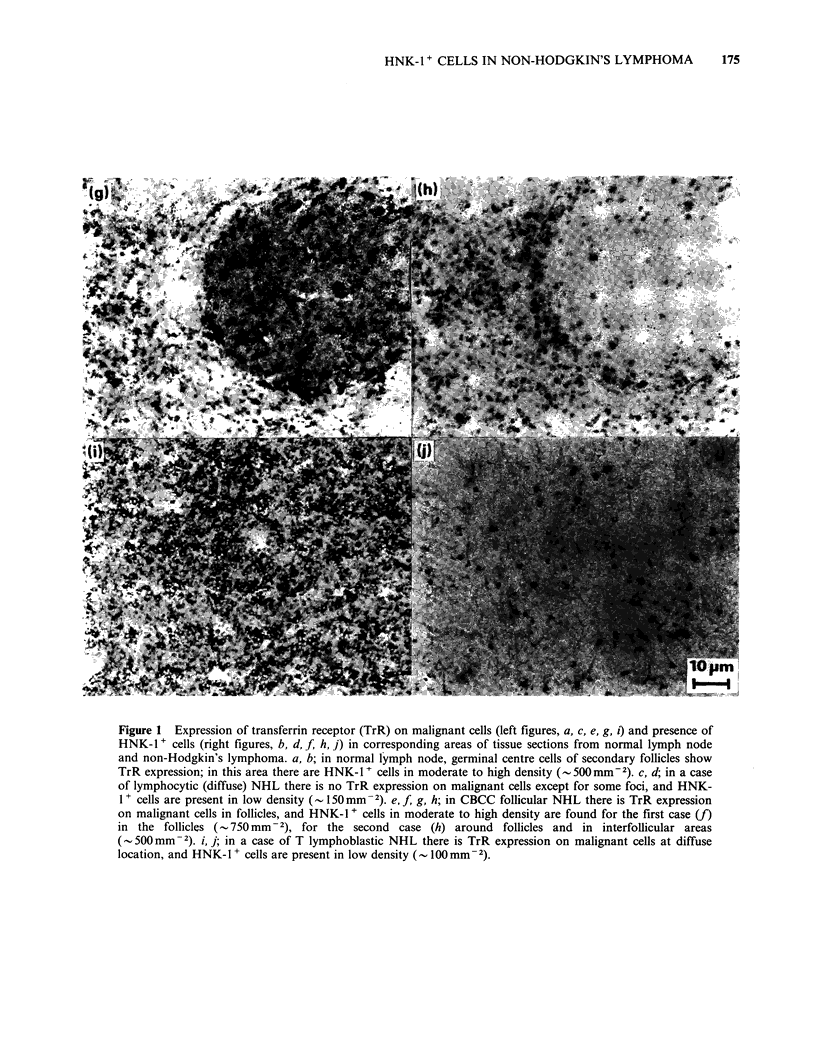

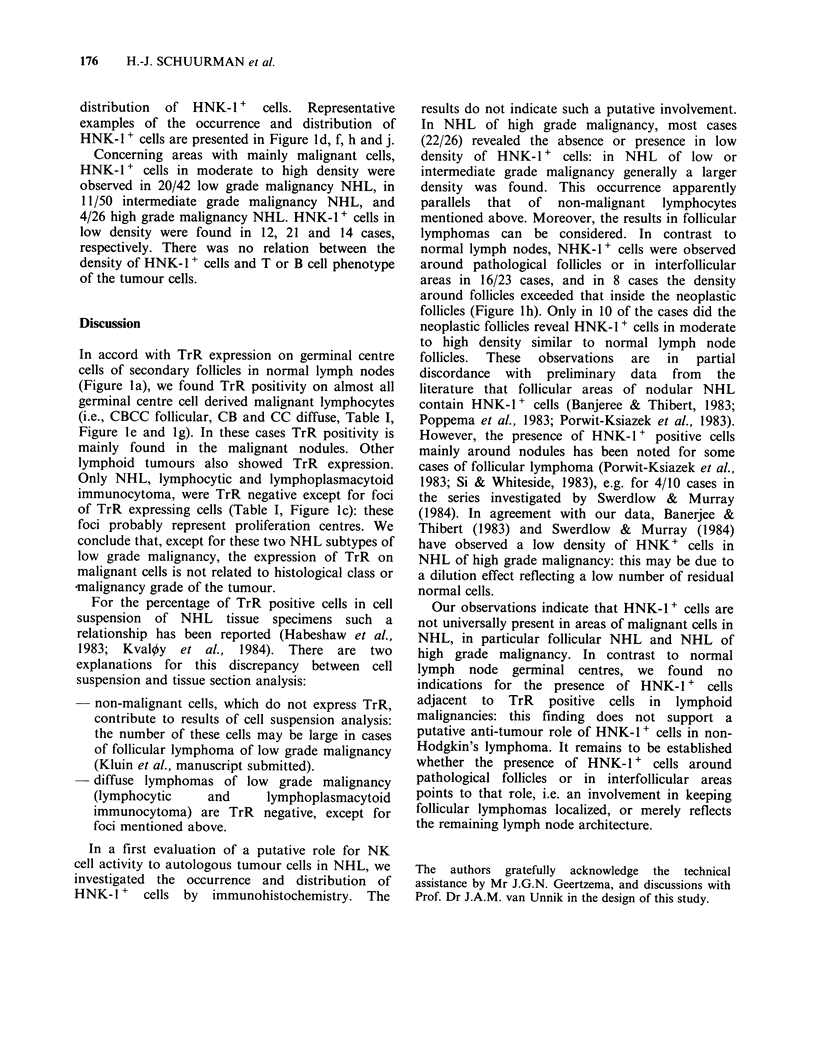

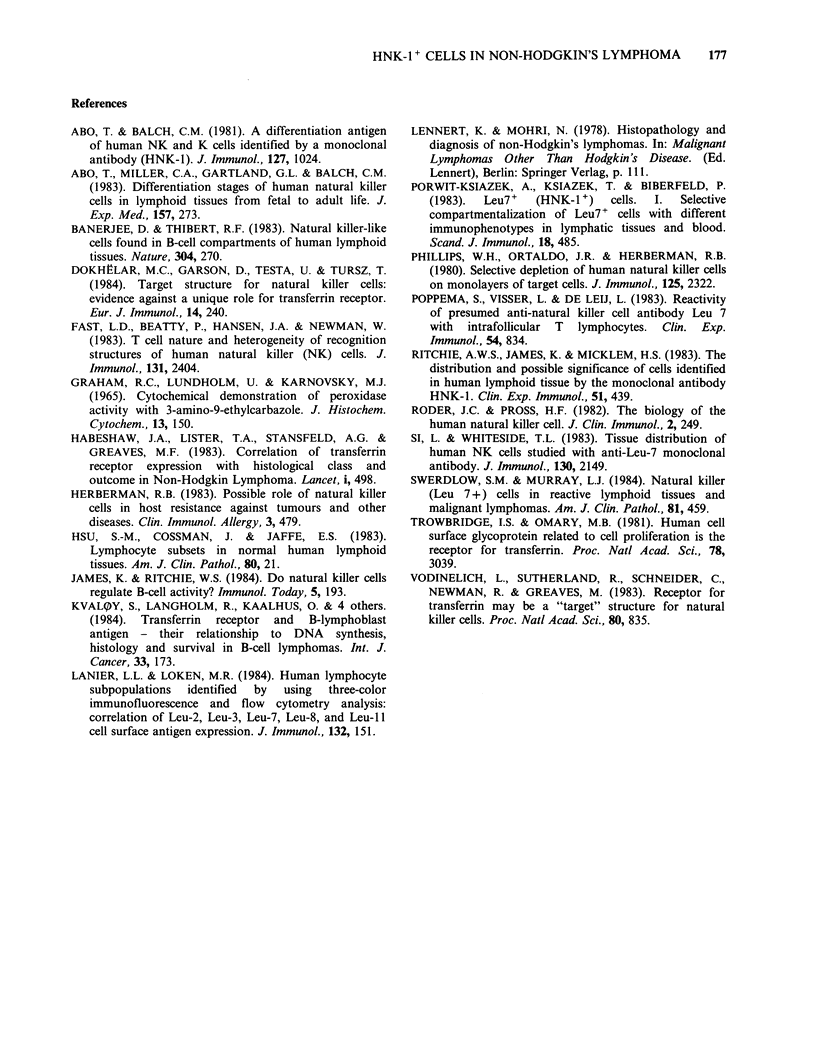


## References

[OCR_00442] Abo T., Balch C. M. (1981). A differentiation antigen of human NK and K cells identified by a monoclonal antibody (HNK-1).. J Immunol.

[OCR_00447] Abo T., Miller C. A., Gartland G. L., Balch C. M. (1983). Differentiation stages of human natural killer cells in lymphoid tissues from fetal to adult life.. J Exp Med.

[OCR_00453] Banerjee D., Thibert R. F. (1983). Natural killer-like cells found in B-cell compartments of human lymphoid tissues.. Nature.

[OCR_00464] Fast L. D., Beatty P., Hansen J. A., Newman W. (1983). T cell nature and heterogeneity of recognition structures of human natural killer (NK) cells.. J Immunol.

[OCR_00470] GRAHAM R. C., LUNDHOLM U., KARNOVSKY M. J. (1965). CYTOCHEMICAL DEMONSTRATION OF PEROXIDASE ACTIVITY WITH 3-AMINO-9-ETHYLCARBAZOLE.. J Histochem Cytochem.

[OCR_00476] Habeshaw J. A., Lister T. A., Stansfeld A. G., Greaves M. F. (1983). Correlation of transferrin receptor expression with histological class and outcome in non-Hodgkin lymphoma.. Lancet.

[OCR_00487] Hsu S. M., Cossman J., Jaffe E. S. (1983). Lymphocyte subsets in normal human lymphoid tissues.. Am J Clin Pathol.

[OCR_00496] Kvaløy S., Langholm R., Kaalhus O., Michaelsen T., Funderud S., Foss Abrahamsen A., Godal T. (1984). Transferrin receptor and B-lymphoblast antigen--their relationship to DNA synthesis, histology and survival in B-cell lymphomas.. Int J Cancer.

[OCR_00503] Lanier L. L., Loken M. R. (1984). Human lymphocyte subpopulations identified by using three-color immunofluorescence and flow cytometry analysis: correlation of Leu-2, Leu-3, Leu-7, Leu-8, and Leu-11 cell surface antigen expression.. J Immunol.

[OCR_00523] Phillips W. H., Ortaldo J. R., Herberman R. B. (1980). Selective depletion of human natural killer cells on monolayers of target cells.. J Immunol.

[OCR_00528] Poppema S., Visser L., De Leij L. (1983). Reactivity of presumed anti-natural killer cell antibody Leu 7 with intrafollicular T lymphocytes.. Clin Exp Immunol.

[OCR_00516] Porwit-Ksiazek A., Ksiazek T., Biberfeld P. (1983). Leu 7+ (HNK-1+) cells. I. Selective compartmentalization of Leu 7+ cells with different immunophenotypes in lymphatic tissues and blood.. Scand J Immunol.

[OCR_00534] Ritchie A. W., James K., Micklem H. S. (1983). The distribution and possible significance of cells identified in human lymphoid tissue by the monoclonal antibody HNK-1.. Clin Exp Immunol.

[OCR_00540] Roder J. C., Pross H. F. (1982). The biology of the human natural killer cell.. J Clin Immunol.

[OCR_00544] Si L., Whiteside T. L. (1983). Tissue distribution of human NK cells studied with anti-Leu-7 monoclonal antibody.. J Immunol.

[OCR_00549] Swerdlow S. H., Murray L. J. (1984). Natural killer (Leu 7+) cells in reactive lymphoid tissues and malignant lymphomas.. Am J Clin Pathol.

[OCR_00554] Trowbridge I. S., Omary M. B. (1981). Human cell surface glycoprotein related to cell proliferation is the receptor for transferrin.. Proc Natl Acad Sci U S A.

[OCR_00560] Vodinelich L., Sutherland R., Schneider C., Newman R., Greaves M. (1983). Receptor for transferrin may be a "target" structure for natural killer cells.. Proc Natl Acad Sci U S A.

